# Effect of Host Species on the Distribution of Mutational Fitness Effects for an RNA Virus

**DOI:** 10.1371/journal.pgen.1002378

**Published:** 2011-11-17

**Authors:** Jasna Lalić, José M. Cuevas, Santiago F. Elena

**Affiliations:** 1 Instituto de Biología Molecular y Celular de Plantas, Consejo Superior de Investigaciones Científicas–Universidad Politécnica de Valencia, València, Spain; 2 The Santa Fe Institute, Santa Fe, New Mexico, United States of America; University of Toronto, Canada

## Abstract

Knowledge about the distribution of mutational fitness effects (DMFE) is essential for many evolutionary models. In recent years, the properties of the DMFE have been carefully described for some microorganisms. In most cases, however, this information has been obtained only for a single environment, and very few studies have explored the effect that environmental variation may have on the DMFE. Environmental effects are particularly relevant for the evolution of multi-host parasites and thus for the emergence of new pathogens. Here we characterize the DMFE for a collection of twenty single-nucleotide substitution mutants of *Tobacco etch potyvirus* (TEV) across a set of eight host environments. Five of these host species were naturally infected by TEV, all belonging to family *Solanaceae*, whereas the other three were partially susceptible hosts belonging to three other plant families. First, we found a significant virus genotype-by-host species interaction, which was sustained by differences in genetic variance for fitness and the pleiotropic effect of mutations among hosts. Second, we found that the DMFEs were markedly different between *Solanaceae* and non-*Solanaceae* hosts. Exposure of TEV genotypes to non-*Solanaceae* hosts led to a large reduction of mean viral fitness, while the variance remained constant and skewness increased towards the right tail. Within the *Solanaceae* hosts, the distribution contained an excess of deleterious mutations, whereas for the non-*Solanaceae* the fraction of beneficial mutations was significantly larger. All together, this result suggests that TEV may easily broaden its host range and improve fitness in new hosts, and that knowledge about the DMFE in the natural host does not allow for making predictions about its properties in an alternative host.

## Author Summary

Mutations are the raw material on which natural selection operates to optimize the fitness of populations. The occurrence of selection and its strength depend on the effect that mutations may have on the survival and reproduction of individuals: mutations can be lethal, deleterious, neutral, or beneficial. Thus, determining how many mutations belong to each of these categories is of importance for predicting the evolutionary fate of a population. For emerging infectious diseases, this distribution determines the likelihood that a pathogen crosses the species barrier and successfully infects a new host. We characterized such distributions across a panel of alternative hosts for a plant virus and found that fitness effects of individual mutations varied across hosts in an unpredictable way and that many mutations considered deleterious in the natural host may turn out to be beneficial in other hosts.

## Introduction

The emergence of new epidemic viruses is a critical issue for public health and economic welfare [1]–[7]. Virus emergence is a complex, multilevel problem that results from a combination of ecological and genetic factors [5]–[8]. The increasing threats imposed by emerging and re-emerging viruses make it even more urgent to predict whether and when virus populations replicating in their reservoir hosts will acquire the ability to successfully infect individuals of a new host species, adapt to it and, eventually, turn into an epidemic. To make such predictions we must first identify the factors determining why some viruses, like *Hepatitis C virus*, *Human immunodeficiency virus* type 1 (HIV-1), *Influenza A virus* or *Cucumber mosaic virus* have been so successful in causing pandemics whereas other viruses such as SARS coronavirus, *Ebola virus*, *Hantan virus*, or *Cocoa swollen shoot disease virus* produced outbreaks limited in time and space. A pre-requisite for viral emergence is the existence of standing genetic variation within the reservoir host that enables successful virus replication within naïve hosts after spillover by chance [2], [3], [8]. As a first approximation, and neglecting the effect of genetic drift, the frequency of these host-range mutants in the reservoir population will directly depend on the equilibrium between (*i*) the rate at which they are produced and (*ii*) the fitness effects they may have in the reservoir host.

If host-range mutations are deleterious in the reservoir host, their frequency will be low and thus the likelihood of emergence will be low as well, whereas if they are neutral or perhaps even beneficial, their frequency will increase, which will in turn increase the chances of emergence. RNA viruses are characterized not only by extremely high mutation rates [9], but also by short generation times and large population sizes [3], [8]. For these reasons RNA viruses have a high evolutionary potential and are over-represented among emerging viruses. Regarding fitness effects, extensive data have shown that host-range mutants have high fitness in the new host but pay fitness penalties in the reservoir host [10]–[13]. This fitness trade-offs should also preclude the evolution of generalist, multi-host viruses [11], [13]–[15]. Antagonistic pleiotropy is often called to explain the existence of such fitness trade-offs [11], [13]. However, an alternative, although not mutually exclusive, mechanism promoting host specialization is the accumulation of neutral mutations in the genes that are not necessary in a given host but are essential in alternative hosts, making these mutations deleterious in the alternative host environment [14], [15].

Therefore, to predict the probability of a virus to infect new hosts, it is necessary to characterize the distribution of mutational fitness effects (DMFE) on its primary hosts as well as on potential new hosts. DMFE across hosts show the fraction of all possible mutations that may be beneficial in new hosts and reveal their fitness effects in the primary host. DMFE have been characterized in recent years for a handful of single-stranded DNA [16], [17] and RNA viruses [16], [18]–[20] in their primary hosts. All these studies but one [18] took a similar experimental approach to the characterization of DMFEs. In all cases, site-directed mutagenesis was performed on infectious clones, generating collections of random single-nucleotide substitution mutants. The fitness of these mutants was then measured by means of competition experiments against the parental non-mutated virus. In [18] (and in some experiments described in [16]), an undetermined number of mutations were fixed by genetic drift in the absence of purifying selection (Muller's ratchet). Three commonalities can be found in these studies [21], which we will briefly summarize. First, all viruses examined show very low tolerance to mutation, as demonstrated by a large fraction of lethal mutations (between 20% and 40%). Second, for non-lethal mutations, the mean fitness loss associated to a single nucleotide substitution is about 10%. Third, DMFEs characterized are left-skewed (i.e., containing more negative values than the Gaussian distribution) and leptokurtic (i.e., comprising less central values than the Gaussian and having longer tails). Accordingly, the probability density functions that better fitted the data were from the heavy-tailed family (Log-normal or Weibull) or highly skewed ones (Gamma or Beta). Still, probably due to the overwhelming amount of work associated with these studies, the effect of host heterogeneity on the properties of DMFE have not been experimentally addressed; with the exception of the work done by Van Opijnen et al. [22] with HIV-1. However, this study was limited to a few single nucleotide-substitution mutations that were not randomly scattered along the viral genome but concentrated in a regulatory non-coding region.

The situation that we have just described in the context of emerging viruses is a particular case of a more general biological problem: the extent to which a phenotype (here viral fitness) is determined by the interaction between the genotype and the environment (here the host species), or the *G*×*E* interaction [23]. Understanding how genotype and environment interact to determine the phenotype and fitness has been a central aim of ecology, genetics, and evolution. Therefore, it should also be central for the epidemiology and evolution of infectious diseases; even more so in light of the reasons given above. The fate of genetic variation in populations depends on the form of the *G*×*E* interactions [24], [25] and, for instance, a change in the rank order of genotypic fitness in different environments may support a balanced polymorphism [25]. Despite this centrality, not much is known about the extent and underlying form of *G*×*E* interactions. Previous attempts to answer these questions suffer from one or another weakness (e.g., non-random samples of mutations taken from standing variation formerly filtered by selection, unknown number of mutations, traits of unclear relationship with fitness, etc.) [26]. To overcome these problems, Remold and Lenski [26] proposed using a collection of mutant genotypes that differ from the wildtype in a single and well defined mutation. Mutational fitness effects should further be evaluated in environments not previously experienced by the organism. By applying this simple experimental design to the bacterium *Escherichia coli*, these authors found that *G*×*E* interactions were quite common even for genotypes that differed by only one mutation and across environments that differed in a single component.

In this study, we sought to study how different host species affect the parameters describing the DMFE for a plant RNA virus, *Tobacco etch potyvirus* (TEV). Furthermore, we were interested in testing whether single point mutations are sufficient to give rise to *G*×*E* interactions in simple and compacted RNA genomes. To do so, we randomly selected 20 single-nucleotide substitution mutants from the collection previously described in Carrasco et al. [20]. Then, we quantified the absolute fitness (i.e., Malthusian growth rate) of all these mutants in eight different host species and characterized the parameters describing the DMFE and how they varied across hosts. Furthermore, we evaluated the amount of observed variability that was explained by genetic differences among viral genotypes, by differences among host species and, more interestingly, by the non-linear interaction between these two factors (e.g., the genotype-by-environment variance). In nature, TEV infects five of these hosts (*Nicotiana tabacum*, *Nicotiana benthamiana*, *Solanum lycopersicum*, *Capsicum annuum*, and *Datura stramonium*), all belonging to the same plant family, the *Solanacea*. The other three species are not TEV natural hosts, although they are experimentally susceptible to systemic infection. They belong to two plant families, the *Asteraceae* (*Helianthus annuus*) and the *Amaranthaceae* (*Gomphrena globosa* and *Spinacea oleracea*). Both the *Solanaceae* and the *Asteraceae* are within the Asterids, while the *Amaranthaceae* are not [27].

## Results

### Characterization of the DMFE on different hosts

**Figure 1:**
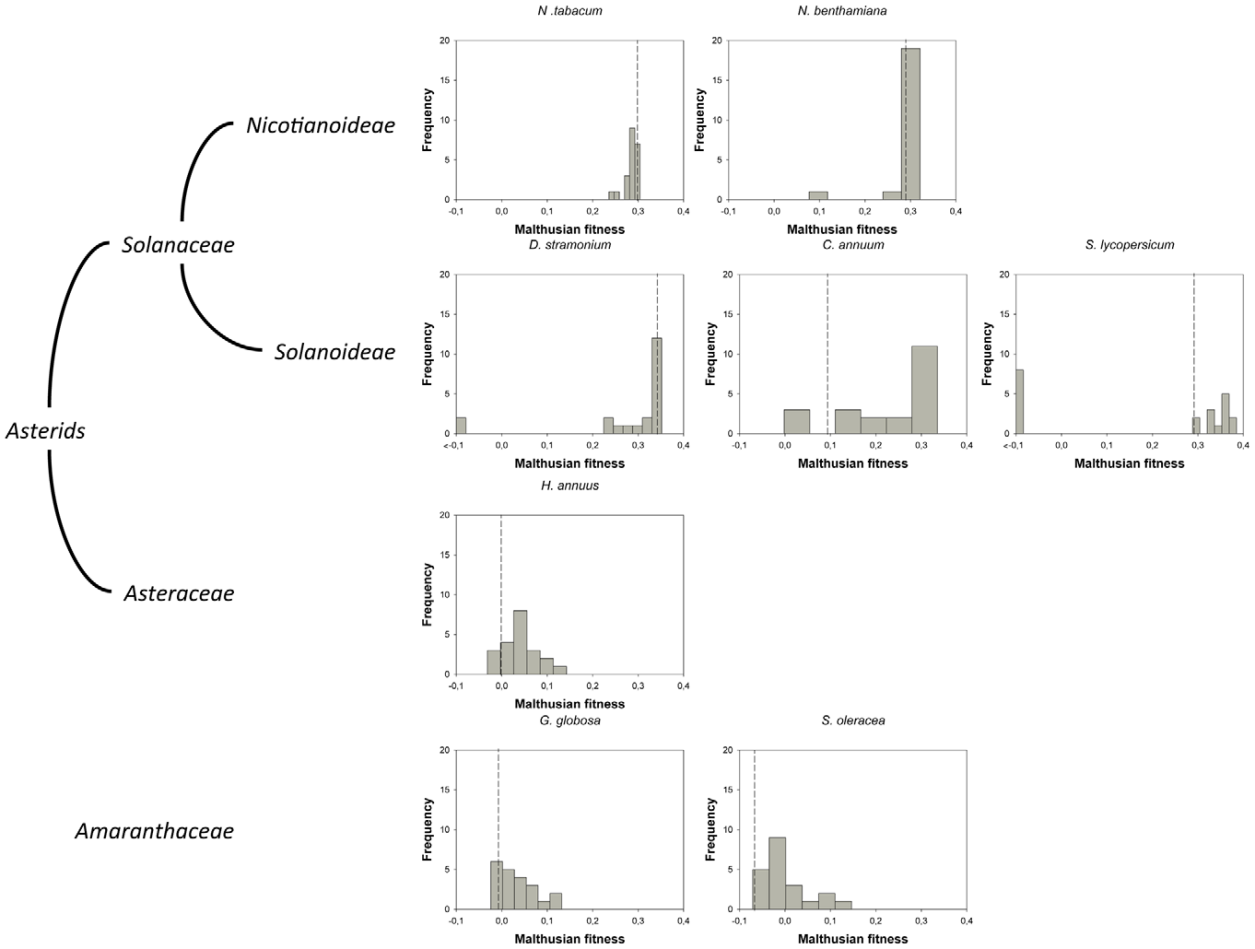
DMFEs across different host species. Host species belong to the taxonomic families *Solanaceae*, *Asteraceae* and *Amaranthaceae*. The first two families belong to the Asterids class. In nature, TEV is found infecting members of the *Solanaceae* family. The ancestral isolate used in this study was obtained from and subsequently passed in *N. tabacum* plants. Lethal mutations (which have a Malthusian fitness of −∞) are indicated in the histograms with <−0.1 fitness values. The vertical dashed lines represent the fitness value of the wildtype genotype in each host.

For this study, we have used a collection of 21 TEV genotypes (20 mutants plus the wildtype) drawn from a larger collection of mutants obtained by Carrasco *et al*. [20]. Each mutant contained a single nucleotide change whose position and substitution were chosen at random. In 14 cases, the mutation resulted in an amino acid substitution (Table 1). Our set of mutants consisted in changes that were randomly dispersed throughout the TEV genome (Table 1). Selected mutants were all viable in the natural host *N. tabacum*. The absolute fitness effects of these genotypes were evaluated in eight susceptible host species. The observed DMFEs for the 21 genotypes in all eight hosts are shown in Figure 1. A quick look at these histograms suggests that in the natural host *N. tabacum* and in its close relative *N. benthamiana* (both species belong to the same genus of the *Nicotianoideae* subfamily) most mutants have absolute fitness indistinguishable from or below the value of the wildtype (indicated by the vertical dashed line; enumerated in Table 2). Indeed, the average absolute fitness values for all mutant genotypes on these two hosts were significantly smaller than the values estimated for the wildtype (Table 2; one-sample *t*-tests, *P*≤0.019 in both cases). Also supporting this excess of deleterious effects, the distributions had significant negative skewness values (Table 2; *t*-test comparing to the Gaussian null expectation, *P*<0.001 in both cases). The average absolute fitness effect of all genotypes together was undistinguishable in these two hosts (Mann-Whitney test, *P* = 0.232). Both distributions are also significantly leptokurtic (Table 2; *t*-test comparing to the Gaussian null expectation, *P*<0.001 in both cases), indicating that many mutations have mild fitness effects and, therefore, the DMFEs are more peaked than expected for a Gaussian distribution. When the absolute fitness of the different TEV mutants was evaluated in hosts whose genetic relatedness to *N. tabacum* decreased, while still belonging to the *Solanaceae* (*Solanoideae* subfamily: *D. stramonium*, *C. annuum* and *S. lycopersicum*), the average value of the distributions did not shift significantly compared to *Nicotianoideae* (Mann-Whitney test, *P* = 0.348). In addition, it remained skewed towards the left tail, that is, the values were smaller than the median of the distribution (Table 2; *t*-test, *P*≤0.026). In *D. stramonium* and *S. lycopersicum*, a few mutations were lethal (see below the arguments supporting the lethality of these mutants), thus making the distributions even more negatively skewed. The change in shape of DMFE noticeably affected the kurtosis parameter. In the three *Solanoideae* hosts DMFEs have no significant kurtosis (Table 2; *t*-tests, *P*≥0.195 in all cases), and thus they are effectively mesokurtic (e.g., Gaussian-like). In general, DMFE dramatically change in several aspects within non-*Solanaceae* hosts. First, the distribution mean shifts towards lower values; a comparison of absolute fitness values between *Solanaceae* and non-*Solanaceae* hosts indicates that the difference is highly significant (Mann-Whitney test, *P*<0.001). Second, the distributions become positively skewed, although the asymmetry was significant only for *S. oleracea* (Table 2; *t*-test, *P* = 0.008). Positive skewness means that the tail of the distribution containing fitness effects higher than the mean is significantly heavier than the negative tail. This finding is particularly interesting when observed that the fitness of the wildtype is always in the negative tail of the distribution.

**Table 1:** TEV genotypes used in this study and some of their properties.

Genotype	Protein	Location	Nucleotide substitution	Amino acid change	Polarity change
DQ986288, wild-type isolate					
PC2	P1	158	U→G	F→C	apolar→polar
PC6	P1	375	A→G	L→M	
PC7	P1	475	A→C	K→Q	basic→polar
PC12	P1	872	A→C	M→L	
PC19	HC-Pro	1503	A→G	synonymous	
PC22	HC-Pro	1655	A→G	N→S	
PC26	HC-Pro	2119	A→U	synonymous	
PC40	P3	3238	T→C	synonymous	
PC41	P3	3406	C→A	Q→K	polar→basic
PC44	P3	3468	U→G	synonymous	
PC49	CI	4418	G→C	S→T	
PC60	CI	5349	U→C	synonymous	
PC63	6K2	5582	A→G	K→R	
PC67	NIa-VPg	6012	U→G	I→M	
PC69	NIa-VPg	6044	C→A	T→N	
PC70	NIa-VPg	6197	U→G	M→R	apolar→basic
PC72	NIa-VPg	6251	U→C	F→S	apolar→polar
PC76	NIa-Pro	6519	U→C	synonymous	
PC83	NIb	7315	A→G	I→V	
PC95	NIb	8501	A→C	E→A	acid→polar

**Table 2:** Parameters describing the DMFE shown in Figure 1 and number of mutations classified as lethal, deleterious, neutral, and beneficial on each host.

	Mean	Median	Std. deviation	Skewness	Kurtosis	Lethal	Deleterious	Neutral	Beneficial
*N. tabacum*	0.280	0.283	0.016	−1.974***	4.608***	0	6	14	0
*N. benthamiana*	0.267	0.277	0.050	−3.949***	16.879***	0	10	10	0
*D. stramonium*	0.307	0.322	0.040	−1.566**	1.364	2	15	3	0
*C. annuum*	0.200	0.260	0.116	−1.037*	−0.389	0	0	9	11
*S. lycopersicum*	0.338	0.349	0.029	−0.768	0.062	8	0	2	10
*H. annuus*	0.026	0.020	0.043	0.527	0.579	0	0	15	5
*G. globosa*	0.019	0.010	0.041	0.997	0.561	0	0	17	3
*S. oleracea*	−0.018	−0.039	0.053	1.479**	1.915	0	0	17	3

*t*-test significance levels for skewness and kurtosis:

*0.05>*P*≥0.01,

**0.01>*P*≥0.001;

***P<0.001.

To further expand the analyses of the data shown in Figure 1, we compared the absolute fitness of each mutant to that of the wildtype TEV on each host using the bootstrap method described in [18]. Based on the bootstrap results, mutations were classified into lethal, deleterious (i.e., significantly smaller absolute fitness than wildtype), neutral, and beneficial (i.e., significantly larger absolute fitness than wildtype) on each alternative host (Table 2). The analysis of this contingency table shows that there is a significant heterogeneity in the distributions of discrete mutational classes among hosts (χ^2^ = 163.262, 21 d.f., *P*<0.001). However, this heterogeneity is entirely driven by the differences among TEV absolute fitness in *Solanaceae* hosts (χ^2^ = 96.161, 12 d.f., *P*<0.001), but not among non-*Solanaceae* hosts (χ^2^ = 0.891, 6 d.f., *P* = 0.989). Indeed, if a new contingency table is constructed by grouping hosts into *Solanaceae* and non-*Solanaceae*, a significant heterogeneity is observed among the two host classes (χ^2^ = 37.884, 3 d.f., *P*<0.001). These results are explained by the shift from more neutral mutations in the two *Nicotianeae* towards more beneficial and lethal in the three *Solanoideae*, while the three non-*Solanaceae* species had similar counts of neutral and beneficial mutations. Interestingly, neutral and non-neutral cases were evenly distributed among synonymous and nonsynonymous mutations for all hosts (Fisher's exact test, *P*≥0.131 in all hosts). In recent years, increasing evidence supports the notion that, for compacted RNA genomes, synonymous mutations are not necessarily neutral mutations [20], [28]. This observation is most likely due to the overlapping nature of many viral genes, the existence of secondary RNA structures essential for regulating gene expression, the adaptation to the host's codon usage bias, and the pressure for evading RNAi-based host defenses.

The above classification of viable mutants into deleterious, neutral or beneficial depends on whether their fitness values deviates significantly from that of the wildtype TEV in the bootstrap test. However, given the statistical uncertainties inherent to our measurements, it is difficult to distinguish between small-effect mutations and lack of fitness effects. For the *Solanaceae*, relative fitness values<−0.03 were generally significantly deleterious, whereas mutations were assigned to the beneficial class if they had relative fitness >0.05 as in *S. lycopersicum*, although the threshold for *C. annuum* rose up to >0.2. For the non-*Solanaceae*, in general, mutations were considered as beneficial if they had relative fitness values >0.05. However, since the concept of neutrality depends on the effective population size [29], modeling the continuous DMFE rather than their discretization, at length, is to be more informative. In the next section we will address this problem.

Failed inoculation experiments and lethal mutations produce the same apparent result: a lack of viral accumulation in the inoculated plants. To rule out the possibility that the putative lethal mutations observed in *D. stramonium* and *S. lycopersicum* are just a succession of failed inoculation experiments, we applied the following statistical argument. First, we evaluated our rate of failure to produce an infection when starting the experiment with viruses that are viable in each host species. In the case of *D. stramonium*, two mutants were assigned to the class of lethals. Out of 171 *D. stramonium* plants inoculated with viable viruses, 72 plants were infected and thus the failure rate was 1–72/171 = 0.579 per inoculation event. After nine trials (corresponding to the number of replicates per mutant and per host species), the probability of failing all cases should be 0.579^9^ = 0.007. Therefore, in a sample of 21 genotypes, we expect less than one case (21×0.007 = 0.153) to be erroneously assigned to the category of lethal mutations. Similarly, in the case of *S. lycopersicum*, where eight mutants were putatively lethal, 72 out of 117 plants inoculated with viable viruses were infected, which represents a failure rate of 0.385 per inoculation experiment. From this, we expect (21×0.385^9^ = 0.004) much less than one case to be classified as lethal but resulting from multiple inoculation failures. Therefore, on these grounds, we are confident that the mutations classified as lethal on these two hosts were really so.

### Fit of empirical DMFE to theoretical probability density functions

Next, we sought to determine which of several competing statistical models better describes the observed DMFEs. Following previous analyses of the DMFE for RNA viruses [16], [18], [19], [20], we evaluated the goodness-of-fit of distributions sharing the property of asymmetry and with heavy tails to the empirical DMFEs observed in each host. Lethal mutations were excluded from the analyses. The probability density functions (pdf) tested were: Exponential, Gaussian, Gamma, Beta, Log-normal, Laplace, Pareto, and Weibull. Nonlinear regression techniques were used to fit models to the data. Table 3 shows the best-fitting model for each host and the relevant parameters describing each distribution, as well as the statistics measuring the goodness of fit (Akaike's weight and *R* ^2^). The Weibull pdf was the model that better described the DMFEs measured in *N. tabacum*, *N. benthamiana*, *D. stramonium*, *S. lycopersicum*, and *G. globosa*. A Weibull pdf is described by two parameters, the scale *λ* and the shape *κ*, related to the expected value of the distribution as 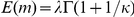, where Γ(·) is the gamma function evaluated at the given argument. However, the Akaike's weight for this pdf is <0.95 in all cases, suggesting that alternative models, or combinations of models, can still contribute to better describe the observed distributions. In the cases of *C. annuum* and *S. oleracea* the pdf that better explained the observed DMFEs were Laplace and Pareto, respectively. These two distributions are from the power-law family. In the case of the Laplace pdf, the expected fitness value is equal to the location parameter *E*(*m*) = *μ*, whereas in the case of the Pareto, the expected value is 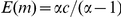, where *α* is the shape parameter and *c* the threshold value. For the two non-Asterids hosts (e.g., *G. globosa* and *S. oleracea*) the expected fitness values were negative, whereas in all other cases the expected fitness values were positive and in the range 0.02–0.311.

**Table 3:** Probability distribution models that best describe the observed DMFEs on each host (excluding lethal mutations).

	Model	Parameter estimates^a^	Expected fitness	Akaike's weight^b^	*R* ^2^	*ER* (to second best model)^c^
*N. tabacum*	Weibull	scale *λ* = 0.286±0.000	0.286	0.706	0.988	7.675 (Normal)
		shape *κ* = 33.138±1.433				
*N. benthamiana*	Weibull	scale *λ* = 0.282±0.000	0.274	0.917	0.989	28.924 (Normal)
		shape *κ* = 20.371±0.840				
*D. stramonium*	Weibull	scale *λ* = 0.323±0.002	0.311	0.643	0.849	4.990 (Laplace)
		shape *κ* = 12.992±2.317				
*C. annuum*	Laplace	location *μ* = 0.253±0.010	0.223	0.521	0.842	5.495 (Weibull)
		scale *b* = 0.104±0.019				
*S. lycopersicum*	Weibull	scale *λ* = 0.324±0.004	0.300	0.479	0.873	2.514 (Normal)
		shape *κ* = 5.774±0.785				
*H. annuus*	Laplace	location *μ* = 0.067±0.001	0.020	1.000	0.992	3721.827 (Normal)
		scale *b* = 0.032±0.014				
*G. globosa*	Weibull	scale *λ* = 0.058±0.001	−0.322	0.400	0.992	1.159 (Beta)
		shape *κ* = 1.358±0.046				
*S. oleracea*	Pareto	threshold *c* = 0.829±0.001	−0.024	0.997	0.930	553.409 (Laplace)
		shape *α* = 22.189±1.493				

a ±1 SE of the estimated value.

b The set of pdf models fitted and compared was: Exponential, Normal, Gamma, Beta, Log-normal, Laplace, Pareto, and Weibull.

c *ER*: evidence ratio. In this case, *ER* measures how many times the best fitting model is more likely than the model ranked in second place.

The Akaike's weight informs about which one among a set of competing models is best supported by the data, after ranking them according to their *AIC* values. However, given the uncertainties associated to the small sample size here used (21 TEV genotypes), one may be interested in evaluating how much better performs the best fitting model relative to any other model. To make this analysis, we used an evidence ratio (*ER*) computed as the likelihood of the best model divided by the likelihood of the alternative model of interest [30]. The last column in Table 3 shows the *ER* values computed for models ranked in second place. The Weibull pdf is the best descriptor in five out of eight host species. Hence, one may ask how good a descriptor it is for the three remaining hosts. In the case of *C. annuum*, the Weibull was ranked as the second best fitting, performing only ∼5.5 times worse than the Laplace pdf. For *H. annuus*, the Weibull pdf ranked in third position, with an *ER* = 38609.153, thus providing a much worse fit than the Laplace pdf. Finally, in the case of *S. oleracea* the Weibull pdf ranked in seventh position, with an *ER* = 190935.254, indicative of a very poor fit compared to the best fitting Pareto pdf.

### The phylogenetic distance between natural and naïve hosts influence the location and shape of DMFE

**Figure 2:**
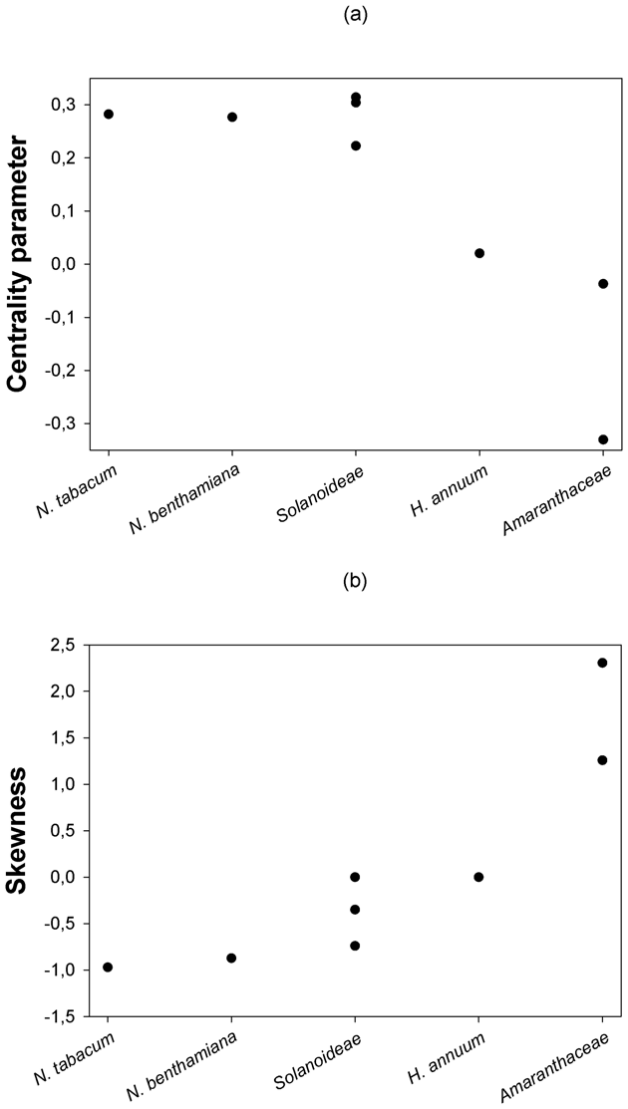
Changes in the centrality and shape parameters of the DMFE with increasing genetic distance among hosts. (a) The centrality parameter of the best fitting pdf shifts from positive to negative Malthusian fitness, indicating that the average effect of single mutations is stronger as the host genetic relatedness with the natural host *N. tabacum* decreases. (b) Distributions become more positively skewed with increasing host genetic distance from *N. tabacum*, suggesting that more mutations have positive effect in the new hosts.

Next, we sought to evaluate whether the location and shape characteristics of the DMFE were affected by the genetic relationship between the hosts. Figure 2a shows that a statistically significant negative correlation (Spearman's *r_S_* = −0.798, 6 d.f., *P* = 0.018) exists between the expected centrality parameter of the DMFE, *E*(*m*) (taken from Table 3), and the ranked phylogenetic distance of each host to the natural one; *N. tabacum*. This negative correlation indicates that the average absolute fitness decreases as the host becomes more and more distant from the one to which the virus was originally adapted. By contrast, a significant positive correlation has been observed between the skewness of the DMFE and host's phylogenetic distance from the natural one (Figure 2b; Spearman's *r_S_* = 0.877, 6 d.f., *P* = 0.004). This result is congruent with the above observation that the skewness of the DMFE shifts from negative to positive as hosts become more phylogenetically distant from the natural one. The phylogenetic distance did not significantly affect the variance and kurtosis of the distributions (in both cases Spearman's *r_S_*≤0.569, 6 d.f., *P*≥0.153).

### Contribution of *G*×*E* interactions to TEV absolute fitness

Model I in Table 4 shows the GLM analysis of the absolute fitness data using host species and TEV genotype as random factors. First, there is a highly significant difference among TEV genotypes in their absolute fitness. This is in agreement with previous analyses of the larger collection of genotypes from which these 20 were drawn [20]. However, only ∼4% of total observed variability is explained by genetic differences among TEV genotypes. There is also a highly significant effect of the host species on viral fitness, which explains ca. 26% of the observed variability in absolute fitness. Finally, and more interestingly from the perspective of predicting emerging viral infections by using information about fitness effects in natural hosts, the *G*×*E* interaction term is also highly significant, and explains ca. 67% of the observed variability in absolute fitness. This significant interaction means that we cannot accurately predict a particular genotype's absolute fitness in a given host from the main effects, thus adding an unpredictability component to viral emergence. Finally, it is worth noting that only 2.76% of the observed variance remained unexplained by the model and was used as error variance in the computation of the different variance components.

**Table 4:** Two generalized lineal models testing the effect of TEV genetic background (*G*), host species (*E*), and their interaction (*G*×*E*).

Source of variation	χ^2^	d.f.	*P*	Variance component^a^	Percentage of variance^b^
*Model I* (*AIC* c = −2328.299)					
*G* (TEV genotype)	2783.062	20	<0.001	4.48×10^−3^	4.29%
*E* (Host species)	6467.415	7	<0.001	2.73×10^−2^	26.13%
*G*×*E*	7282.589	140	<0.001	6.99×10^−2^	66.82%
*Model II* (*AIC* = −2412.799)					
*G* (TEV genotype)	2783.062	20	<0.001	4.32×10^−3^	4.17%
*Host class*	1371.172	1	<0.001	8.56×10^−3^	8.25%
*E* (species within *Host class*)	3177.883	6	<0.001	1.81×10^−2^	17.47%
*G*×*E*	7282.589	140	<0.001	6.99×10^−2^	67.33%

Both variables were treated as random sources.

a Maximum-likelihood estimators.

b For *Model I*, computed using a value of error variance equal to 2.88×10^−3^, which is equivalent to a 2.76% of unexplained variance. For Model II, computed with an error variance 2.88×10^−3^ (2.77%).

c Akaike information criterion.

To account for the fact that hosts are not independent but phylogenetically related, we fitted a more complicated model to the data (Model II in Table 4). This alternative model treated the host species as a binary factor; belonging to one of two classes (*Solanaceae* vs. non-*Solanaceae*). Then, host species were nested within these two classes and the *G*×*E* component was evaluated by looking the significance of the interaction between hosts within classes and TEV genotype. This model has an appreciably lower *AIC* value than the Model I and thus should be taken as a better one, although the conclusions do not qualitatively depart from those reached from the simpler model (Model I): the genetic component only explains a minor fraction of observed fitness variance whereas most of it is explained by the *G*×*E* interaction term.

### The causes of *G*×*E*

A significant *G*×*E* interaction can be produced by two non-mutually exclusive mechanisms [26]. First, pleiotropic effects may change the rank order of mutations across environments (e.g., a mutation beneficial in one environment may not be so in an alternative one). Second, while still retaining the rank order of fitness effects, *G*×*E* can also be generated by altering the genetic component of phenotypic variance (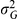) across hosts. To evaluate the contribution of these two mechanisms to the observed *G*×*E*, we run two different analyses.

**Figure 3:**
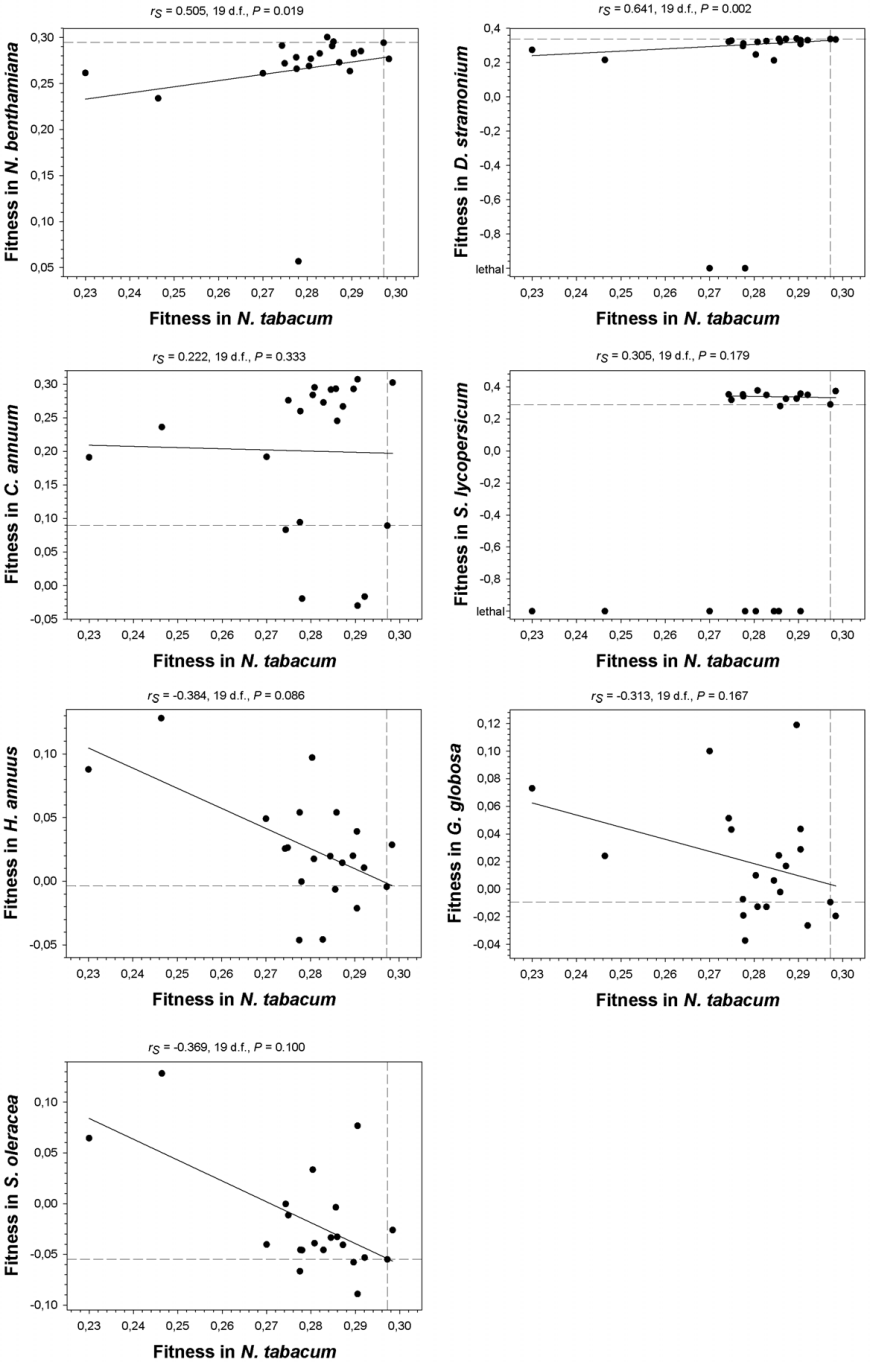
Relationship between fitness in *N. tabacum* and in the seven alternative hosts. Spearman's non-parametric correlation coefficients and their statistical significance are shown above each plot. The non-parametric test was chosen given its robustness against extreme data points. Dashed lines represent the fitness of the wildtype TEV in the corresponding hosts. The solid lines are only inserted to illustrate the overall trend.

As a first statistical test, we computed Spearman's rank correlation coefficients between absolute fitness values in the primary host *N. tabacum* and the values estimated on each alternative host (Figure 3). Lethal mutations were assigned to the lowest rank. A negative correlation would indicate negative or antagonistic pleiotropy (e.g., mutations change the strength and sign of their effects on different hosts), whereas a positive correlation would indicate positive pleiotropy. Interestingly, the correlations were positive for all the *Solanaceae* hosts (although only reached significance in two cases, *N. benthamiana* and *D. stramonium*). By contrast, for the three non-*Solanaceae* hosts the correlation coefficients had negative non-significant values. We used the frequency of discrete mutational signs on each host class to construct a contingency table, and applied a Fisher's exact test to confirm that the difference in correlation signs among host classes was significant (*P* = 0.029) despite the small sample size. Furthermore, the shift from negatively skewed DMFE (excess of deleterious effects) in the *Solanaceae* to positively skewed distributions (excess of beneficial effects) in the non-*Solanaceae* described above is also consistent with antagonistic pleiotropy. Therefore, from these analyses we concluded that antagonistic pleiotropy contributed to generate *G*×*E* when the new host species are phylogenetically distant from the natural host (i.e., outside the plant family), but not when host species belong to the same family. Nevertheless, this conclusion needs to be qualified because the most extreme cases of antagonistic pleiotropy are mutations that were viable in *N. tabacum* but lethal in *D. stramonium* and *S. lycopersicum*, all being from the same family.

A non-significant correlation test, however, cannot be taken as an evidence of a lack of pleiotropic effects across hosts. For instance, one can imagine a situation in which, in a given host, some mutations may have negative pleiotropic effects, some others positive ones and some even being independent on the host. In such situation, the correlation would turn out to be non-significant while still some mutations may be pleiotropic. To overcome this drawback, we performed a second statistical test based on the frequency of mutations that changed the sign of its fitness effects (compared to that of the wildtype TEV) across hosts. For each mutation on each host, we recorded whether fitness was lower (negative sign) or higher (positive sign) than the wildtype TEV. Then we counted the number of cases for which the sign changed between the primary host, *N. tabacum*, and each alternative one. If a mutation has the same sign both in the primary and in the alternative hosts, it is considered not to be pleiotropic. By contrast, if sign changes, then it is considered as pleiotropic. Under the null hypothesis of no excess of pleiotropic effects, mutations would distribute evenly across both categories. Departures from this null hypothesis were evaluated using Binomial tests. Only in *N. benthamiana* (*x* = 2) and *D. stramonium* (*x* = 4) the number of observed mutations with putative pleiotropic effects was not significantly larger than expected under the null expectation (probability of having *x* or more cases of pleiotropic mutations than expected by sheer chance: *P*<0.001 and *P* = 0.006, respectively). By contrast, the number of mutations whose fitness effects switched signs were significantly larger than expected by chance in all other hosts: *x* = 18 in *C. annuum* (*P*>0.999), *x* = 19 in *S. lycopersicum* (*P*>0.999), *x* = 14 in *H. annuus* (*P* = 0.942), 15 in *G. globosa* (*P* = 0.979), and 17 in *S. oleracea* (*P*>0.999). Therefore, this second test of antagonistic pleiotropy confirmed the conclusions drawn from the Spearman's correlation test. Moreover, it showed that antagonistic pleiotropy also made an important contribution to the fitness variability observed in the two hosts (*C. annuum* and *S. lycopersicum*) in which no overall tendency was observed in Figure 2.

Next, to evaluate the importance of changes in genetic variance, 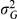, for absolute fitness as a source of *G*×*E* we computed it for each of the eight host species. Table 5 shows the estimates of 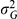, of error variance (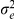) as well as the broad sense heritability (*H* ^2^) that indicates the percentage of total phenotypic variance explained by genetic differences among TEV genotypes. For the five *Solanaceae* hosts, 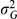 ranged from 0.051 to 0.115, with an average value of 0.083, and 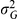 explaining >95% of the observed phenotypic variance. By contrast, 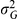 within the non-*Solanacea* hosts was significantly smaller (Mann-Whitney test, *P* = 0.036), with an average value of ∼0.002. Besides, for these hosts only ca. 25% of phenotypic variance for absolute fitness was explained by genetic differences among TEV mutants. Henceforth, from these analyses we conclude that changes in genetic variance for absolute fitness contributed to the observed *G*×*E* only when comparing phylogenetically distant hosts.

**Table 5:** Maximum likelihood estimators for the variance components of absolute fitness estimated on each host (± variance of the estimator).

Host species	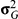 (×10^−2^)	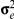 ( ×10^−4^)	*H* ^2^
*N. tabacum*	7.858±0.059	3.524±0.000	0.996
*N. benthamiana*	7.323±0.051	16.052±0.000	0.979
*D. stramonium* a	9.462±0.097	40.160±0.006	0.959
*C. annuum*	5.162±0.028	61.520±0.015	0.894
*S. lycopersicum* a	11.475±0.203	6.204±0.000	0.995
*H. annuus*	0.148±0.000	48.061±0.006	0.236
*G. globosa*	0.109±0.000	47.062±0.006	0.188
*S. oleracea*	0.195±0.000	46.762±0.005	0.294

a Lethal alleles were removed from the computations because they have absolute fitness −∞.

All together, these results suggest that *G*×*E* arises from the combined effect of antagonistic pleiotropy and reductions in genetic variance associated to the shift from hosts that belong to the same family as the natural host to hosts that do not belong to this family.

## Discussion

### Changes in DMFE and the likelihood of crossing the species barrier

New emerging epidemic viruses represent one of the most serious threats to human, animal and crops health [1]–[8]. The problem of viral emergence is complex and depends on the interaction between host's genetics, vectors' abundance, ecology, and virus evolvability. Predicting the potential of a virus to spillover from its natural host reservoir to few individuals of a new host species and successfully establish a productive infection that will trigger a new epidemic seems an insurmountable problem. However, from the perspective of evolutionary genetics, the problem can be simplified by considering that the fate of the viral population entering into the new host depends, in a first instance, on whether it contains genetic variants with a positive fitness value. In other words, a pre-requisite for predicting the ability of a virus to expand its host range is to have information about the distribution of fitness effects associated to mutations (DMFE) across all possible hosts. In this study, we have characterized DMFE across a set of hosts for the plant virus TEV. The host species selected widely ranged in their degree of genetic relatedness with the natural host, *N. tabacum*: from very close relatives (members of the same genus) to members of other genera within the same family, and finally, to species belonging to different families within the same class or even to different classes. We found that the central parameter of the DMFE shifted towards smaller values as the phylogenetic distance of each host from tobacco increased (Figure 2a). The distributions did not just displace; they also changed in shape, moving most of the probability mass from the negative to the positive tails. This means that, on average, the absolute fitness of TEV decreased as hosts became more different from the natural one. However, if the fitness of individual mutant genotypes is expressed relative to wildtype virus, the change in shape means that the number of (conditional) beneficial mutations increases as hosts become more phylogenetically distant from tobacco. This suggests that the number of mutations that may potentially expand TEV host range is large. A similar abundance of host-range mutants was also observed for phage φ6 [12]. In this case, the mutations were concentrated in the P3 gene that encodes for the protein responsible for attaching the virion to the bacterial pili. However, in our case, host-range mutations do not concentrate in any particular gene but were scattered along the genome. Notably, Gaussian fitness landscape models [31] predict an increase in the proportion of beneficial mutations under stressful conditions (here represented by those hosts in which absolute fitness was dramatically reduced).

The shape of DMFE is a critical component of many mathematical models of evolutionary dynamics, including the molecular clock, the rate of genomic contamination by Muller's ratchet, the maintenance of genetic variation at the molecular level, and the evolution of sex and recombination [32]. In more practical terms, characterizing the shape of DMFE is essential for understanding the nature of quantitative genetic variation, here including complex human diseases as well as pathogens virulence [32]. Therefore, it is not surprising that much effort has been recently invested in characterizing the DMFE for many organisms (reviewed in [32]), including several RNA and DNA viruses. Despite differences in the genetic material of these viruses, their sizes and gene contents, the methodology applied has been similar in all cases, namely, generating collections of single-nucleotide substitutions mutants and then characterizing the fitness of each of these mutants relative to the non-mutated parental. In RNA viruses such as bacteriophage Qβ [16], *Vesicular stomatitis virus* (VSV) [19] and TEV [20], over one third of mutations generated unviable viruses, whereas viable mutations reduced fitness, on average, by ∼10% [21]. Regarding the theoretical pdf that better explained these datasets, VSV fitness data conformed to a complex distribution combining a Log-normal and an Uniform pdfs, the original TEV larger dataset was best fitted by a Beta pdf (notice that in [20] fitness was measured as a relative value, which may justify the difference to the Weibull pdf conclusion reached here), and the Qβ DMFE was well described by a Gamma pdf. In the case of DNA phages φX174 [16] and f1 [17] the fraction of unviable mutations was lower (one fifth) but the average effect of viable mutations was almost identical to the one reported for RNA viruses [21]. φX174 best fitting was to the Exponential pdf whereas for f1 the Log-Normal and the Weibull fitted equally well. Taken together, all these results suggested the existence of certain common rules: a large fraction of mutations are lethal or have a large negative fitness effects (displaying the fragility of viral genomes). In addition, DMFE for viruses are highly asymmetric and can be reasonably well described by theoretical pdfs with heavy tails. In a recent study [33], the reason for this generality was grounded into the thermodynamic properties of protein folding, suggesting that the effect of mutations on protein folding and stability was a good explanation for the observed DMFEs. Despite being important for understanding the evolution of a virus in its natural host, these results were, even so, insufficient to understand the likelihood of a virus expanding its host range. Here, we have contributed to cover this lack of knowledge by describing the effect of changing hosts on the properties of DMFE. One of the most striking conclusions from our study is that the fraction of lethal, deleterious, neutral and beneficial mutations, and hence the shape and location of the distributions, radically depends on the host in which the fitness effects of mutations is evaluated, and that this dependence is, itself, conditioned by the phylogenetic distance among hosts. Furthermore for host species belonging to the same family as the primary host, the Weibull pdf fitted best (or second to best for *C. annuum*) model to describe DMFE, although for hosts outside the family this model is the best only in one out of three cases (Table 3).

Martin and Lenormand [31] proposed three possible outcomes for the DMFEs measured in permissive *vs*. stressful environments: (*i*) conditional expression means that some mutations have a detectable fitness effect in some environments but are neutral in others, (*ii*) conditional average means that the average mutational effect differs between the two types of environments and (*iii*) conditional variance, meaning that variance in mutational effects changes between the two types of environments. In a survey of DMFE across benign and stressful environments for organisms as diverse as the fungi *Saccharomyces cerevisiae* and *Cryptococcus neoformans*, the nematode *Caenorhabditis elegans*, and the fruitfly *Drosophila melanogaster*, Martin and Lenormad [31] found that stressful conditions tend to inflate the variance of the DMFE while leaving the central value of the distributions almost unaffected. These results contrast with those reported here: for TEV, DMFE evaluated in stressful hosts (the non-*Solanaceae*) had lower average (Figure 2a) and more positive skewness (Figure 2b) than in permissive hosts (the *Solanaceae*), while no significant effects on variance were observed. Furthermore, we found that some mutations that were neutral in the natural host had reduced absolute fitness in alternative ones. Therefore, our data contain all three possible outcomes proposed by Martin and Lenormand [31], thus suggesting that their expectations were somewhat simplistic.

A compelling idea of the phylogenetic constraints for a virus jumping the host species barrier resides in the argument that the more closely related the primary host and the new host are, the greater are the chances for a successful spillover [34]. There are good mechanistic reasons that argue for it; if the ability to recognize and infect a host cell is important for cross-species transmission, then phylogenetically related species are more likely to share related cell receptors and defense pathways. However, others support the opposed view based on the observation that spillovers have occurred between hosts that can be either closely or distantly related, and no rule appears to predict the susceptibility of a new host [35]. Whether or not phylogenetic relatedness between reservoir and new hosts may be a factor for host switching, the rate and intensity of contact may be even more critical. Viral host switches between closely related species (e.g., species within the same genera) may also be limited by cross-immunity to related pathogens [2]; paraphrasing Holmes and Drummond [35] “although a species might be exposed to a novel pathogen, they might, through a combination of shared common ancestry and good fortune, already posses a sufficient immune response to prevent the infection from being established”. Our results shed some light into this debate: certainly the absolute fitness of a virus may be reduced when colonizing a new host, especially those distantly related ones, but the fraction of mutations that may be beneficial in this new host also increases with phylogenetic distance between the new host and the reservoir.

### Pleiotropy and changes in genetic variance as sources of *G*×*E* interactions

The existence of *G*×*E* interactions in determining fitness has been well established for many organisms, however, many of these studies used genotypes that differed in a large and unknown number of mutations [23], [36]–[39], making unclear whether *G*×*E* depended on single plasticity genes or on the quantitative contribution of multiple genes. Furthermore, in many examples, these studies used genotypes sampled from natural populations and thus have been filtered out by natural selection. Interestingly, our data demonstrate that single random nucleotide substitutions are sufficient to produce a significant *G*×*E* interaction. Mutations involved in significant *G*×*E* were scattered along the genome and they were randomly chosen irrespective of their fitness effects, provided they were viable in the primary host *N. tabacum*. Thus, we can conclude that phenotypic plasticity of TEV is not associated to the expression of any particular gene but results from the contribution of different genes. The concordance of these results with those previously reported by Remold and Lenski [26] for the bacterium *E. coli* and using knockout mutations suggests that the contribution of individual mutations to *G*×*E* is a general norm. In the context of emerging viral infections, the existence of a significant *G*×*E* interaction means that by knowing the absolute viral fitness in the natural host informs us little about what it may be in an alternative one, thus minimizing our ability to predict which genetic variants may be relevant for expanding TEV host-range.

Two non-mutually exclusive explanations can be brought forward to explain the existence of *G*×*E*: a change in the rank order of mutational effects across hosts (i.e., pleiotropy) and a change in the magnitude of the genetic variance but without changing the rank order. The evolutionary implications for these two mechanisms are different. Changes in genetic variance imply that the relative influence of selection and drift on the fate of mutations depends on the host. Exposure to hosts where the genetic variance in absolute fitness effects is low minimizes the efficiency by which selection operates either removing deleterious alleles or fixing beneficial ones and thus enhances the role of drift. By contrast, changes in rank order imply that selection favor different mutations in different hosts thus driving to a balanced polymorphism and specialization. We have assessed the extent to which these two possibilities may contribute to the observed *G*×*E* and found that both indeed coexist. Antagonistic pleiotropy does not contribute significantly to *G*×*E* when the novel host is closely related to the natural one, however, it becomes an important factor when hosts are distantly related (Figure 3). Similarly, genetic variance for absolute fitness was similar within *Solanaceae* hosts, but approximately one order of magnitude smaller for hosts outside the *Solanaceae*. Therefore, we conclude that the observed *G*×*E* interaction can be explained both by antagonistic pleiotropy and by changes in the genetic component of variance. Previous studies with *E. coli* showed that *G*×*E* was mainly explained by changes in genetic variance but not by changes in the rank order of fitness effects across environments [26]. However, other authors found that the contribution of new mutations to *G*×*E* for fitness traits in *D. melanogaster* was mostly via antagonistic pleiotropy [40].

The significant positive pleiotropy observed between absolute fitness in the natural host *N. tabacum* and in two closely related alternative ones (*N. benthamiana* and *D. stramonium*) suggests that mutations ameliorate aspects of the virus interaction with host factors that may be common to all three hosts but not to the other hosts. By contrast, the antagonistic pleiotropy observed between absolute fitness in *N. tabacum* and in the non-*Solanaceae* hosts suggests that TEV may be interacting with different host factors and that the improved interaction with tobacco may led to less efficient interaction with an orthologous factor, if available, in the alternative hosts. In this regard, many examples exist in the plant virology literature showing that host-range mutations have negative pleiotropic effects in the natural host (reviewed in [8], [41]). An illustrative example is the interaction between the VPg protein of other potyviruses and the host translation initiation factor eIF4E [42], [43]. Translation of the viral genomic RNA into the polyprotein depends upon the correct attachment between VPg and eIF4E. Mutations in eIF4E have been identified as the cause of the *Potato virus Y* (PVY) resistant phenotype of pepper cultivars. However, PVY overcomes the resistance by fixing amino acid changes in the central domain of VPg that reconstitutes the correct binding. These mutants pay a fitness cost in the non-resistant pepper.

### Concluding remarks

Here we have shown for the first time how DMFE for an RNA virus vary across hosts. Our results suggest that the location of the DMFE moves towards smaller values as the phylogenetic distance to the natural host increases. In parallel, the distribution switches from negative to positive skewness, indicating that the probability of potential beneficial mutations increases along with host genetic distance. Similarly, we have found that the virus genotype and the host species interact in a non-linear manner to determine viral fitness. Both pleiotropic effects and reductions in genetic variance contribute to generate this genotype-by-host interaction. The implications of these observations for our understanding of emerging viral infections are multiple, but basically all hint on the unpredictability at the level of individual mutations: in the light of information collected on the primary host one can not anticipate which particular viral genotypes will be more likely to emerge. However, antagonistic pleiotropy still leaves some room for predictability at the level of classes of mutations: beneficial mutations, as a class, in the natural host *may* become deleterious in an alternative one, or vice versa.

## Materials and Methods

### Virus genotypes

For this study, a subset of 20 mutants non-lethal in *N. tabacum* (Table 1) was randomly chosen from a larger collection used in a previous study [20]. A plasmid containing the TEV genome, pMTEV [44], generously gifted by Dr. J.A. Daròs, was used to generate both the wildtype virus and the mutant genotypes. Single-nucleotide substitution mutants were generated by site-directed mutagenesis using QuikChange II XL Site-Directed Mutagenesis Kit (Stratagene) as described in [20] and following the manufacturer's instructions. The kit incorporates PfuUltra high fidelity DNA polymerase that minimizes the introduction of undesired mutations. The uniqueness of each mutation was confirmed by sequencing an 800 bp fragment encompassing the mutagenized nucleotide.

Infectious RNA of each genotype was obtained by *in vitro* transcription after BglII linearization of the corresponding plasmid as described in [45]. The infectivity of each RNA genotype was tested by inoculating five *N. tabacum* plants. All TEV genotypes were confirmed to be infectious on *N. tabacum*.

### Host species

Eight host species previously described as susceptible to TEV systemic infection (VIDE database; pvo.bio-mirror.cn/refs.htm) were chosen for these experiments. Five hosts belong to the *Solanaceae* family: *N. tabacum*, *N. benthamiana*, *D. stramonium*, *C. annuum*, and *S. lycopersicum*. The first two belong to the same genus of the *Nicotianoideae* subfamily whereas the other three belong to the *Solanoideae* subfamily [27]. One host, *H. annuus*, pertains to the *Asteraceae* family. Both *Solanaceae* and *Asteraceae* are classified as Asterids [27]. The remaining two hosts, *G. globosa* and *S. oleracea* belong to the family *Amaranthaceae*. The three plant families are Eudicots [27].

### Inoculation experiments

All hosts were at similar growth stages when inoculated in order to minimize infectivity error due to possible variation in defense response to infection with developmental stage. All inoculations were done in a single experimental block. Nine plants per host per TEV genotype (9×8×21 = 1512) were inoculated by rubbing the first true leaf with 5 µL containing 5 µg RNA *in vitro* transcript of the virus and 10% carborundum (100 mg/mL). *Solanaceae* hosts show clear symptoms when infected and thus visual inspection was enough for determining infection. Nonetheless, some randomly chosen asymptomatic *Solanaceae* plants were subjected to RT-PCR for detection of infection as described in [46]. None was positive in this test. In the case of the non-*Solanaceae* hosts, symptoms were not recognizable and thus, infection was confirmed by RT-PCR.

Ten days post-inoculation (dpi), the whole infected plant, except the inoculated leaf, was collected. The whole tissue was frozen in liquid nitrogen and ground with mortar and pestle.

### RNA purification and virus quantification

An aliquot of approximately 100 mg of grounded tissue was taken and mixed with 200 µL of extraction buffer (0.2 M Tris, 0.2 M NaCl, 50 mM EDTA, 2% SDS; pH 8). An equal volume of phenol∶chloroform∶isoamylic alcohol (25∶25∶1) was added, thoroughly vortexed and centrifuged at 14000 g for 5 min at 25°C. Ca. 160 µL of the upper aqueous phase were mixed with 80 µL of a solution containing 7.5 M LiCl and 50 mM EDTA and incubated overnight on ice at 4°C for RNA precipitation. The precipitated RNAs were centrifuged at 14000 g for 15 min at 4°C, washed once with 70% ice-cold ethanol, dried in a SpeedVac (Thermo) and resuspended in 30 µL of DEPC-treated ultrapure water. RNA concentration was measured spectrophotometrically and the samples were diluted to a final concentration of 50 ng/µL.

Within-plant virus accumulation was measured by absolute RT-qPCR using external standard [47]. Standard curves were constructed using five serial dilutions of TEV RNA produced by *in vitro* transcription and diluted in RNA obtained from the corresponding healthy host plant species. Samples were grouped by hosts and quantity of viral RNA was calculated using the corresponding standard curve.

RT-qPCR reactions were performed in 20 µL volume using One Step SYBR PrimeScript RT-PCR Kit II (TaKaRa) following the instructions provided by the manufacturer. The primers forward TEV-CP 5′-TTGGTCTTGATGGCAACGTG and reverse TEV-CP 5′-TGTGCCGTTCAGTGTCTTCCT amplify a 71 nt fragment within the TEV CP cistron. CP was chosen because it locates in the 3′ end of TEV genome and hence would only quantify complete genomes but not partial incomplete amplicons. Each RNA sample was quantified three times in independent experiments. Amplifications were done using the ABI PRISM Sequence Analyzer 7000 (Applied Biosystems). The thermal profile was as follows: RT phase consisted of 5 min. at 42°C followed by 10 s at 95°C; and PCR phase of 40 cycles of 5 s at 95°C and 31 s at 60°C. Quantification results were examined using SDS7000 software v. 1.2.3 (Applied Biosystems).

### Statistics

Absolute fitness was estimated as Malthusian growth rate per day, according to expression 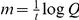, where *Q* is the number of pg of TEV RNA per 100 ng of total plant RNA quantified at *t* = 10 dpi.

Unless otherwise indicated, all statistical tests were performed using SPSS version 19. Generalized linear models (GLM) were used to explore the effect of the different factors on TEV fitness. We assumed that *m* was distributed either as a Gaussian pdf or as a more stretched Gamma pdf. In both cases an identity link function was used. No qualitative differences were observed between the results obtained with these alternative distributions. Results reported will be those obtained using the Gaussian model.
